# Tumor necrosis factor-α, TNF receptor, and soluble TNF receptor responses to aerobic exercise in the heat

**DOI:** 10.1016/j.cytox.2020.100033

**Published:** 2020-07-12

**Authors:** Eliott Arroyo, Joseph A. Laudato, Brandon M. Gibson, Cody S. Dulaney, Jeremiah A. Vaughan, Brittany N. Followay, Ellen L. Glickman, Adam R. Jajtner

**Affiliations:** Exercise Science Program, Kent State University, Kent, OH, USA

**Keywords:** Tumor necrosis factor-alpha, Heat stress, Aerobic exercise, Monocytes, TNF receptors

## Abstract

The purpose of this study was to evaluate the effects of aerobic exercise in the heat on circulating concentrations of tumor necrosis factor (TNF)-α, soluble TNF receptors (STNFR1&2), and surface expression of TNFR1&2 on monocyte subpopulations. Twelve recreationally active Caucasian men (24.4 ± 3.4 yrs.; 180.0 ± 6.8 cm; 81.5 ± 8.0 kg; 47.2 ± 4.8 mL·kg^−1^·min^−1^) completed an exercise protocol in three environmental conditions: high temperature/low humidity [HTLH; 35 °C, 20% relative humidity (RH)]; high temperature/moderate humidity (HTMH; 35 °C, 45%RH); and moderate temperature/moderate humidity (MTMH; 22 °C, 45%RH). Each protocol consisted of a 60-minute cycling trial at 60% VO_2_max, a 15-minute rest, and a time-to-exhaustion trial at 90% VO_2_max (TTE). Blood was sampled before (PRE), immediately after (POST) the 60-minute trial, immediately post-TTE (PTTE), and one-hour post-TTE (REC). Circulating TNF-α and STNFR1&2 were assayed. TNFR1&2 expression on monocyte subsets was measured by flow cytometry on a subset of participants (n = 8). TNF-α area under the curve with respect to increase (AUCi) was greater during HTMH compared to MTMH and HTLH. STNFR1 concentration was greater during HTMH compared to MTMH. With all trials combined, STNFR1 concentration increased from PRE to POST, PTTE, and REC. TNFR1 expression on non-classical monocytes was greater during HTMH compared to HTLH while TNFR2 expression was lower during HTLH compared to both MTMH and HTMH. Data suggest that exercise in the heat increases circulating TNF-α and STNFR1 concentration concomitantly. Furthermore, non-classical monocyte expression of TNFRs are impacted by temperature and humidity during exercise.

## Highlights

• Aerobic exercise in the heat promotes modest increases in plasma TNF-α and STNFR1.
• Increases in TNF-α and STNFR1 are likely driven by changes in core temperature.
• TNFR1 and 2 expression on non-classical monocytes is blunted one hour post-exercise.
• TNFR1 expression on non-classical monocytes is elevated during exercise in the heat.

## 1 Introduction

Aerobic exercise in the heat is associated with decreases in performance [1] as well as elevated stress hormones including cortisol [2], epinephrine and norepinephrine [3]. The additive effect of heat stress during exercise also leads to elevated circulating concentrations of cytokines such as interleukin (IL)-6 [2] and tumor necrosis factor (TNF)-α [3] when compared to exercise in a neutral environment. During strenuous exercise, intestinal blood flow is reduced and gut permeability increases allowing for the infiltration of bacterial lipopolysaccharide (LPS or endotoxin) into circulation [4]. Although mechanisms are in place to neutralize and remove LPS from circulation, prolonged intense exercise in the heat markedly reduces blood flow to the gut leading to compromised epithelial tight junctions and elevated LPS concentration in the blood, termed endotoxemia [4]. This increase in LPS concentration initiates a pro-inflammatory cascade characterized by activation of leukocytes, increases in cytokine concentration, and fever, which can further augment heat stress [5].

TNF-α is rapidly released in response to infection, exposure to LPS, or trauma and has a pivotal role in orchestrating the pro-inflammatory cascade that follows [6]. It has previously been described as a “double-edged sword” given its wide range of both beneficial (e.g. tumor necrosis, tissue regeneration, immunity from infections) and harmful (tumorigenesis, tissue wasting, autoimmunity, heart disease) effects [7]. Primarily produced by monocytes, macrophages, and other cells of monocytic lineage, TNF-α binds with high affinity to two cognate transmembrane receptors: TNF receptor (TNFR) 1 and TNFR2 (CD120a and CD120b, respectively) [8]. The pleiotropic effects of TNF-α are possible due to the presence of these receptors on nearly all cell types. TNFR1 can be found on all human cell types [7] and predominantly mediates inflammation and tissue degeneration; whereas TNFR2 is found on vascular endothelial cells and immune cells and is mainly associated with tissue regeneration and immune modulation [8], [9]. Both receptors can be found in circulation as soluble TNFR1 (STNFR1) and STNFR2 following cleavage from the cell surface by TACE (TNF-α converting enzyme), also known as disintegrin and metalloproteinase 17 (ADAM17) [6]. Ectodomain shedding of these receptors serves a protective role against deregulated TNF-α signaling by both sequestering and inhibiting the ligand in circulation and by limiting the number of signal-competent receptors on the cell surface [9].

Significant increases in TNF-α concentration have been observed following prolonged bouts (2–3 h) of aerobic exercise [10], [11]. However, aerobic exercise of shorter duration (90 min or less) is not sufficient to elicit this response. To this end, studies comparing the effects of 40 min [12] and 90 min [3], [13] cycling in thermoneutral versus hot conditions observed a significant increase in TNF-α following the hot condition and no change in the thermoneutral condition. These findings suggest that exercise alone is an insufficient stimulus for TNF-α release and an elevation of core temperature during physical stress is a key determinant in the inflammatory response to exercise.

In 2010, Ziegler-Heirbrock and colleagues [14] proposed a nomenclature defining three types of human monocytes based on the expression levels of LPS coreceptor CD14 and the Fcγ receptor III CD16: classical, intermediate, and non-classical monocytes. Classical monocytes express high levels of CD14 but no CD16 (CD14^++^CD16^−^), intermediate monocytes show a high level of CD14 and low CD16 (CD14^++^CD16^+^), and the non-classical monocytes express low CD14 and high CD16 (CD14^+^CD16^++^). These monocyte subsets vary in their cytokine production, surface marker expression, and function [15]. Specifically, evidence suggests that CD16^+^ monocytes preferentially produce TNF-α [16], though there is little agreement on whether non-classical or intermediate monocytes are the greatest producers of TNF-α [15]. Furthermore, Hijdra and colleagues [17] found that intermediate monocytes have the highest TNFR1 expression among monocyte subsets and non-classical monocytes show the highest TNFR2 expression.

Military personnel, fire fighters, and athletes are often required to perform vigorous physical tasks in extreme heat and humidity. Exercise in hot conditions is associated with cardiovascular drift [18], increased circulating stress hormones and catecholamines [19], as well as increased reliance on carbohydrates [20] when compared to exercise in thermoneutral conditions. Whether as a result of environmental heat stress and/or physical exertion, hyperthermia has been shown to influence various aspects of the immune response [21], modifying the production of several inflammatory cytokines and magnifying the degree of exercise-induced immunological changes [3], [12], [13]. However, the extent to which exercise in hot conditions affects monocyte expression of TNFRs and circulating levels of STNFRs has not been investigated. Elucidation of the effects of exercise in the heat on the TNF system has important health implications. Plasma levels of TNF-α [22] and STNFRs [23] are elevated in persons with heat stroke, and an imbalance between inflammatory and anti-inflammatory signaling may lead to inflammation-induced injury and immunosuppression [24].

Few studies have evaluated the impact of heat stress on the TNF response to aerobic exercise. Although previous studies have reported elevated TNF-α concentrations during and following exercise in the heat [3], [12], no study, to our knowledge, has investigated the effects of exercise in the heat on STNFR concentration or TNFR expression on monocyte subsets. Therefore, the purpose of this study was to evaluate the combined effects of aerobic exercise and heat stress on circulating concentrations of TNF-α, STNFRs, and surface expression of TNFRs on monocyte subsets.

## 2 Materials and methods

### 2.1 Participants

A total of 15 recreationally active Caucasian men were recruited to participate in this experimental study. Women were excluded due to sex-related differences in the immune response [25], [26], [27] as well as thermoregulation [28], [29]. Three participants were removed from the investigation prior to analysis due to non-compliance with the study protocols. Therefore, a total of 12 participants (24.4 ± 3.4 yrs.; 180.0 ± 6.8 cm; 81.5 ± 8.0 kg; 25.0 ± 3.1 kg·m^−2^; 47.2 ± 4.8 mL·kg^−1^·min^−1^) were included in the analysis. Due to limited resources, TNFR1 and TNFR2 expression on monocyte subsets was measured on the final eight participants to complete the study (25.0 ± 3.4 yrs.; 180.2 ± 7.1 cm; 81.7 ± 9.7 kg; 25.3 ± 3.6 kg∙m^−2^; 47.3 ± 4.9 mL·kg^−1^·min^−1^). Inclusion criteria included age 18–30 yr., free of any physical limitations that may affect performance, and maximal oxygen uptake (VO_2_max) ≥ 3.0 L/min as determined by a graded exercise test. Additionally, all participants were free of any medications, performance enhancing drugs, and any dietary supplements that have antioxidant or recovery properties as determined by a health and activity questionnaire. Using the procedures described by Beck [30], a minimum sample size of ten participants was necessary to achieve a minimum power (1 – *β*) of 0.80 at an alpha level of 0.05. Power calculations were made using G*Power statistical analyses software (Version 3.1.9.2, Düsseldorf, Germany) based on changes in TNF-α reported previously [3], [13].

Following an explanation of all procedures, risks, and benefits, each participant provided his written informed consent prior to participating in this study. The research protocol and the informed consent document were approved by the University’s Institutional Review Board prior to participant enrollment.

### 2.2 Study design

**Fig. 1:**
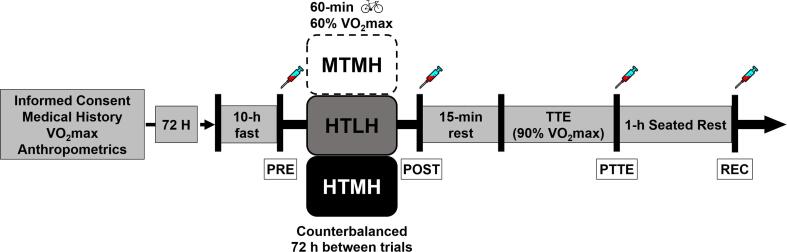
Study Design. Participants completed a VO_2_max assessment and exercise protocols in three conditions: high temperature/low humidity [HTLH; 35 °C, 20% relative humidity (RH)]; high temperature/moderate humidity (HTMH; 35 °C, 45% RH); and moderate temperature/moderate humidity (MTMH; 22 °C, 45%RH). Each exercise protocol consisted of a 60-minute cycling trial at 60% VO2max, a 15-minute rest, and a time-to-exhaustion trial at 90% VO2max (TTE). Blood samples were collected before (PRE) and after (POST) the 60-minute trial; immediately after TTE (PTTE), and one-hour post-TTE (REC). Exercise protocols were completed in a randomized, counterbalanced fashion separated by ≥72 h.

Participants reported to the laboratory on four separate occasions (Fig. 1). The first visit consisted of obtaining informed consent, medical history, anthropometric testing, and a graded exercise test to determine eligibility for the study and to establish workloads. Body mass (±0.1 kg) and height (±0.1 cm) were measured using a physician’s scale (Detecto, Webb City, MO, USA), and body mass index (BMI; kg/m^2^) was calculated as body mass (kg) ÷ height (m)^2^. On the subsequent three visits, participants arrived at the laboratory following a 10-hr fast and completed an exercise protocol in three environmental conditions: high temperature/low humidity [HTLH; 35 °C, 20% relative humidity (RH)]; high temperature/moderate humidity (HTMH; 35 °C, 45% RH); and moderate temperature/moderate humidity (MTMH; 22 °C, 45% RH). Each exercise protocol was performed in an environmental chamber (Cincinnati Sub-Zero, Cincinnati, OH, USA) and consisted of a 60-minute cycling trial at 60% VO_2_max, a 15-minute rest, and a time-to-exhaustion trial at 90% VO_2_max (TTE). Participants were asked to complete the exercise trials while fasted due to the potential blunting effect of protein [31] and carbohydrate [32] on post-exercise cytokine production. Exercise protocols were performed on an electronically braked cycle ergometer (Velotron Dynafit Pro, QUARQ, Spearfish, SD, USA). Blood samples were collected before (PRE) and immediately after (POST) the 60-minute trial; immediately after TTE (PTTE), and one-hour post-TTE (REC). Participants remained in a seated and reclined position in the environmental chamber and were allowed to drink water ad libitum during the one-hour recovery following TTE. Exercise protocols were completed in a randomized, counterbalanced fashion separated by ≥72 h. Participants were asked to drink 3 L of water and to abstain from exercise, tobacco use, and alcohol consumption during the 72 h prior each visit.

### 2.3 Study procedures

#### 2.3.1 Dietary recall

Participants were asked to provide a 24-hour dietary recall prior to each exercise trial while maintaining their regular diet for the duration of the investigation. The United States Department of Agriculture Food Composition Database (USDA Agricultural Research Service, Beltsville, MD, USA version 3.9.5.1) was used to analyze the self-reported dietary recalls for total kilocalorie intake, macronutrient proportions (carbohydrate, protein, and fat), as well as sodium (Na) and potassium (K) intake.

#### 2.3.2 VO_2_max

Participants were fitted with a heart rate (HR) monitor (Polar Electro, Woodbury, NY, USA) and prepared for an incremental test to exhaustion on a cycle ergometer. For the first four stages, workload was set to 50 W and increased in a stepwise fashion by 50 W every 3 min. For the rest of the stages, workload increased by 30 W every 2 min until volitional fatigue. Throughout the test, pulmonary gas exchange data (Parvo Medics TrueOne, Sandy, UT, USA), and heart rate were monitored continuously, while ratings of perceived exertion (RPE; 6–20 scale) were collected at the end of each stage [33]. Participants were considered to have reached maximum capacity if their respiratory exchange ratio (RER) exceeded 1.1, RPE exceeded 17, or a plateau was observed in HR or VO_2_, despite an increased workload. Test termination criteria followed the ACSM guidelines [34]. Once volitional fatigue was reached, VO_2_max was recorded and used to determine the workloads for the experimental trials. Workloads for the experimental trials were determined by plotting average VO_2_ at each stage against intensity and calculating a regression equation.

#### 2.3.3 Core temperature

Upon arrival at the laboratory, participants self-inserted a rectal thermistor (ITP010-11, Nikkiso—Therm Co., Ltd., Japan) 13 cm beyond the anal sphincter to measure rectal temperature (T_re_) during the experimental trials. Thermocouples for T_re_ were continuously monitored using a data logger throughout the trials (Model N543, Nikkiso—Therm Co., Ltd., Japan). Though no sessions were affected, experimental trials would have been terminated if T_re_ reached 39.5 °C.

#### 2.3.4 Blood measurements

Venous blood samples were obtained from the antecubital space using a 20-gauge disposable needle equipped with a Vacutainer tube holder (Becton Dickinson, Franklin Lakes, NJ). For PRE, participants were instructed to lay supine for 15 min prior to the blood draw. For POST, PTTE and REC, blood samples were collected in a seated and reclined position within 5 min of completing exercise or after rest. With the exception of PRE, all blood draws were performed inside the environmental chamber. Blood samples were collected into two K_2_EDTA treated Vacutainer® tubes (Becton Dickinson)—a 10 mL, and a 4 mL tube. The 4 mL K_2_EDTA treated tube was used for complete blood counts and flow cytometry analysis. The blood in the 10 mL tube was subsequently centrifuged (Centrifuge 5810 R, Eppendorf, Hauppauge, NY, USA) at 3000 RPM for 15 min. The resulting plasma samples were placed into separate 1.8 mL microcentrifuge tubes and frozen at −80 °C for later analysis.

#### 2.3.5 Biochemical analysis

Hematocrit, hemoglobin, and complete blood counts with a 3-part differential were analyzed via an automated hematology analyzer (Coulter® AC·T diff 2™, Beckman Coulter, Brea, CA). Plasma volume shifts were calculated using the formula established by Dill & Costill [35]. Plasma concentrations of TNF-α, STNFR1, and STNFR2 were measured via commercially available high-sensitivity enzyme-linked immunosorbent assay (ELISA; R&D Systems, Inc. Minneapolis, MN, USA), per manufacturer’s instructions, using an Epoch™ 2 Microplate Spectrophotometer (BioTek, Winooski, VT, USA). To eliminate interassay variance, all samples for each assay were thawed once and analyzed in the same assay run by a single technician. All samples were analyzed in duplicate with a mean intra-assay variance of 4.46% for TNF-α, 2.79% for STNFR1, and 4.21% for STNFR2.

#### 2.3.6 Cell staining

K_2_EDTA-treated peripheral whole blood (100 μL) was used to identify monocyte subsets and quantify the TNFR1 and TNFR2 expression by direct immunofluorescent labeling of cell surface antigens with mouse and rat anti-human monoclonal antibodies. All antibodies were titrated prior to data collection to determine optimal antibody concentrations. A sample was heat-treated to induce cell death and determine viability dye expression for the exclusion of non-viable cells. Briefly, a 200 µL aliquot of whole blood was transferred to a 12 × 75 mm polystyrene test tube and placed in 65 °C water for 1 min. The sample was then placed in 4 °C armor beads (Lab Armor, LLC, Cornelius, OR, USA) for 1 min before another 200 µL of fresh whole blood was added to the test tube and used for analysis.

Cells were first stained with an amine-reactive fluorescent dye (Zombie Green™, Biolegend, San Diego, CA, USA) and incubated in the dark for 15 min to determine cell viability. After incubation, samples were washed with 1 mL of wash buffer containing 1% fetal bovine serum (FBS; Life Technologies, Grand Island, NY, USA) and 0.1% sodium azide (NaN_3_; VWR, Radnor, PA, USA) in a 1 × phosphate buffered saline (PBS) solution (Life Technologies). Samples were then centrifuged (Centrifuge 5810 R, Eppendorf) at 300*g* for 8 min, and the supernatant was discarded. Nonspecific staining was blocked with Fc receptor blocking solution (Human TruStain FcX™, Biolegend). Phycoerythrin (PE) conjugated anti-CD16 (3G8; Biolegend), peridinin chlorophyll protein complex (PerCP) conjugated anti-CD14 (M5E2; Biolegend), and allophycocyanin (APC) conjugated anti-CD120a (W15099A; Biolegend) and anti-CD120b (3G7A02; Biolegend) were used in the receptor labeling process. Surface staining was performed by adding 25 μL of the appropriate antibody cocktail (Table 1) to the cell suspension and incubating in the dark for 15 min at room temperature. After staining, samples were lysed with 2 mL of RBC Lysis/Fixation solution (Biolegend) and subsequently mixed and incubated in the dark for an additional 12 min. Following incubation, samples were centrifuged and washed once more. Finally, samples were fixed in 300 μL of 2% paraformaldehyde (Santa Cruz Biotechnology, Dallas, TX, USA) in PBS.

**Table 1:** Flow cytometry antibody panel.

	FITC	PE	PerCP	APC
Tube 1	–	–	–	–
	–	–	–	–
Tube 2	Zombie	–		
*Heat Treated*	–	–	–	–
Tube 3	Zombie	CD16	–	–
	–	(3G8)	–	–
Tube 4	Zombie	–	CD14	–
	–	–	(M5E2)	–
Tube 5	Zombie	CD16	CD14	–
	–	(3G8)	(M5E2)	–
Tube 6	Zombie	CD16	CD14	CD120a
	–	(3G8)	(M5E2)	(W15099A)
Tube 7	Zombie	CD16	CD14	CD120b
	–	(3G8)	(M5E2)	(3G7A02)

Antibody (Clone). FITC = Fluorescein isothiocyanate. PE = Phycoerythrin. PerCP = peridinin chlorophyll protein complex. APC = allophycocyanin.

#### 2.3.7 Flow cytometry

**Fig. 2:**
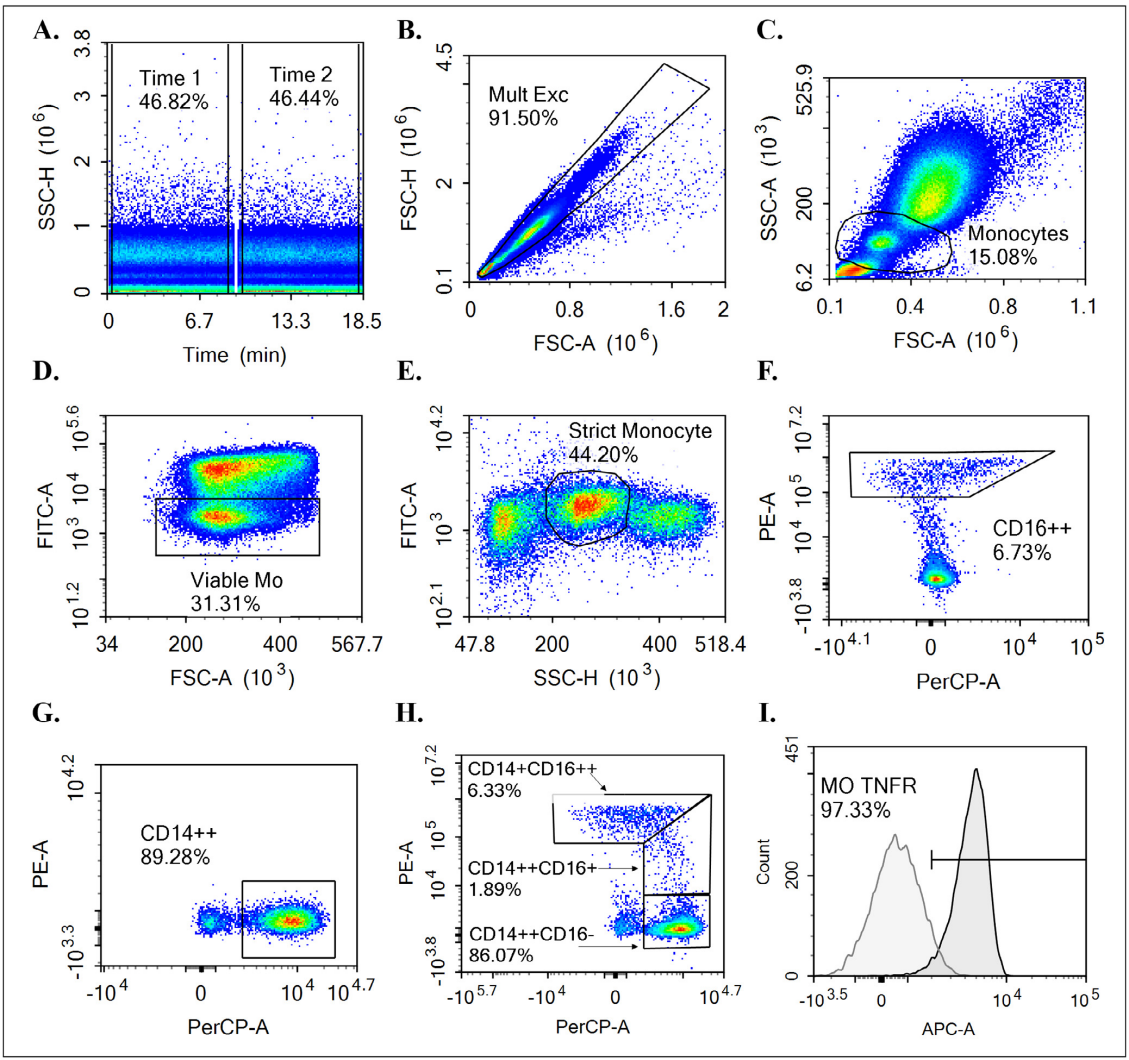
Flow Cytometry Gating Procedure. (A) Samples were initially gated mid flow. (B) Multiplet cells were excluded based on forward scatter (FSC). (C) Generous gating was used to identify monocytes based on FSC/side scatter (SSC) characteristics. (D) Non-viable cells were excluded based on Zombie Green expression on a heat-treated sample. (E) Strict gating was used to isolate monocytes based on SSC of viable cells. Fluorescence-minus-one (FMO) controls were used for CD16 (F) and CD14 (G). (H) Classical (CD14^++^CD16^−^), non-classical (CD14^+^CD16^++^), and intermediate (CD14^+^^+^CD16^+^) monocytes were identified. (I) Finally, tumor necrosis factor receptor (TNFR)1 and TNFR2 expression was assessed on each monocyte subset.

Flow cytometric analysis of stained cells was performed on an ACEA NovoCyte™ Flow Cytometer (ACEA Biosciences, Inc, San Diego, CA), equipped with two lasers providing excitation at 488 and 640 nm, four band pass filters, and ACEA’s NovoExpress™ software. Events were gated based on forward scatter (FSC), side-scatter (SSC), and median fluorescence intensity (MFI) as depicted in Fig. 2. Compensation for fluorescence spillover was achieved through staining of anti-mouse and anti-rat Ig, κ/negative control compensation particles (BD CompBeads, BD Biosciences, San Jose, CA, USA) on a bi-weekly basis. Unstained blood samples and fluorescence-minus-one (FMO) controls were used as negative control for CD120a and CD120b expression. A total of 200 μL were collected for each sample. Monocyte subsets were determined based on the nomenclature proposed by Ziegler-Heitbrock [14] as classical (CD14^++^CD16^−^), intermediate (CD14^++^CD16^+^), and non-classical (CD14^+^CD16^++^) monocytes. Median CD120a and CD120b expression was assessed on each monocyte subset using one-dimensional histograms. Surface expression was reported as MFI. The gating strategy employed is depicted in Fig. 2.

### 2.4 Statistical analysis

Prior to statistical procedures, all data were assessed to ensure normal distribution, homogeneity of variance, and sphericity. If the assumption of sphericity was violated, a Greenhouse-Geisser correction was applied. Cytokine concentration and surface expression were analyzed using separate trial by time mixed-model analyses of variance (ANOVAs) for each dependent variable. Mixed-model regression analysis, with trial and timepoint as fixed factors was used to account for interindividual differences in resting cytokine concentrations and receptor expression. The net area under the curve with respect to increase (AUCi) was also calculated for immunological measures using a standard trapezoidal technique [36] and was assessed using one-way within subjects ANOVAs. Dietary intake during the 24 h prior to each experimental trial was analyzed using one-way, repeated measures ANOVA. T_re_ during exercise trials was analyzed using trial by time repeated measurement ANOVA. In the event of a significant F ratio, the least significant difference (LSD) post-hoc analysis was used for pairwise comparisons. If a significant interaction was observed, follow-up one-way, repeated measures ANOVA and LSD pairwise comparisons were used to determine time effects within each trial and trial effects at each timepoint. Time effects were further analyzed using partial eta squared (η^2^_p_). Interpretations of η^2^_p_ were evaluated according to Cohen [37] at the following levels: small effect (0.01–0.058), medium effect (0.059–0.137), and large effect (>0.138). Significance was accepted at α ≤ 0.05. Data were analyzed using IBM SPSS Statistics for Windows (version 21.0; IBM Corp., Armonk, NY). All data are presented as mean (SD).

## 3 Results

### 3.1 Dietary intake

Dietary intake during the 24 h prior to the experimental trials is presented in Table 2. No main effect for trial was noted for total caloric, protein, carbohydrate, or fat intake during the 24 h prior to each experimental trial. Additionally, no main effect for trial was noted for sodium or potassium intake.

**Table 2:** Dietary intake.

	Caloric Intake (kcal·day^−1^)	Carbohydrate (g·day^−1^)	Fat (g·day^−1^)	Protein (g·day^−1^)	Sodium (mg·day^−1^)	Potassium (mg·day^−1^)
MTMH	2391.7 (370.2)	250.2 (73.8)	100.9 (46.4)	123.7 (45.4)	3387.5 (1323.6)	2137.7 (1206.3
HTLH	2611.9 (626.2)	292.1 (98.7)	114.8 (27.8)	107.7 (21.5)	3870.2 (1651.2)	2019.2 (964.7)
HTMH	2198.8 (610.7)	284.9 (178.2)	84.3 (32.1)	106.6 (38.9)	3397.6 (2112.9)	1692.2 (785.3)

Mean (SD).

### 3.2 Core temperature

**Fig. 3:**
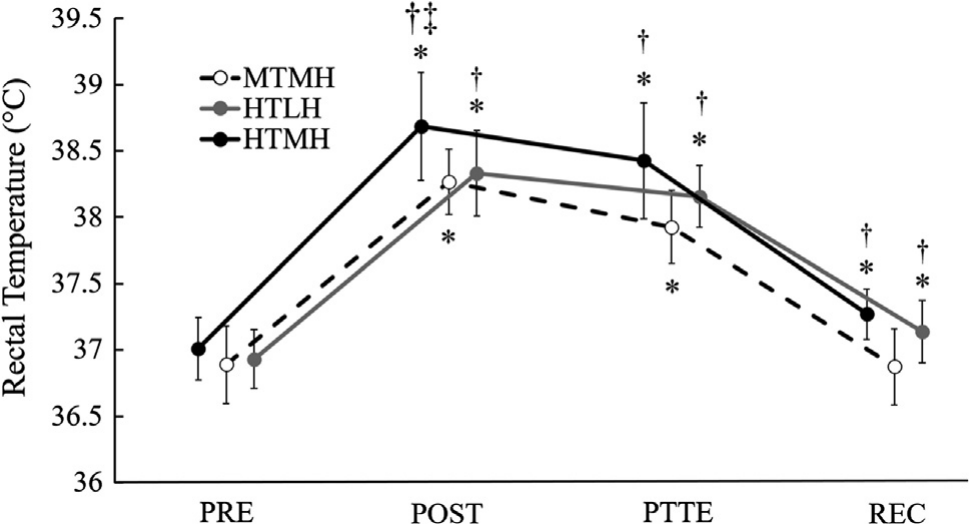
Changes in Rectal Temperature. Trials: HTLH, high temperature/low humidity; MTMH, moderate temperature/moderate humidity; HTMH, high temperature/moderate humidity. *Significant difference from PRE for corresponding trial. † Significant difference from MTMH. ‡ Significant difference from HTLH. Data are presented as mean (SD).

Changes in T_re_ during the experimental trials are shown on Fig. 3. A significant trial × time interaction (F = 3.233, *p* = 0.049, η^2^_p_ = 0.244) was observed for T_re_. For MTMH, T_re_ was highest at POST relative to all other timepoints (*p* < 0.01), remained elevated at PTTE relative to PRE (*p* < 0.01), and returned to pre-exercise values at REC. For both HTLH and HTMH, T_re_ was highest at POST relative to all other timepoints (*p* ≤ 0.03) and remained elevated at PTTE (*p* < 0.01) and REC (*p* ≤ 0.026) relative to PRE (*p* ≤ 0.03). T_re_ at POST was higher during HTMH compared to MTMH (*p* < 0.010) and HTLH (*p* = 0.014). T_re_ at PTTE and REC was higher during HTMH (*p* ≤ 0.006) and HTLH (*p* ≤ 0.007) compared to MTMH.

### 3.3 Plasma volume shifts

**Fig. 4:**
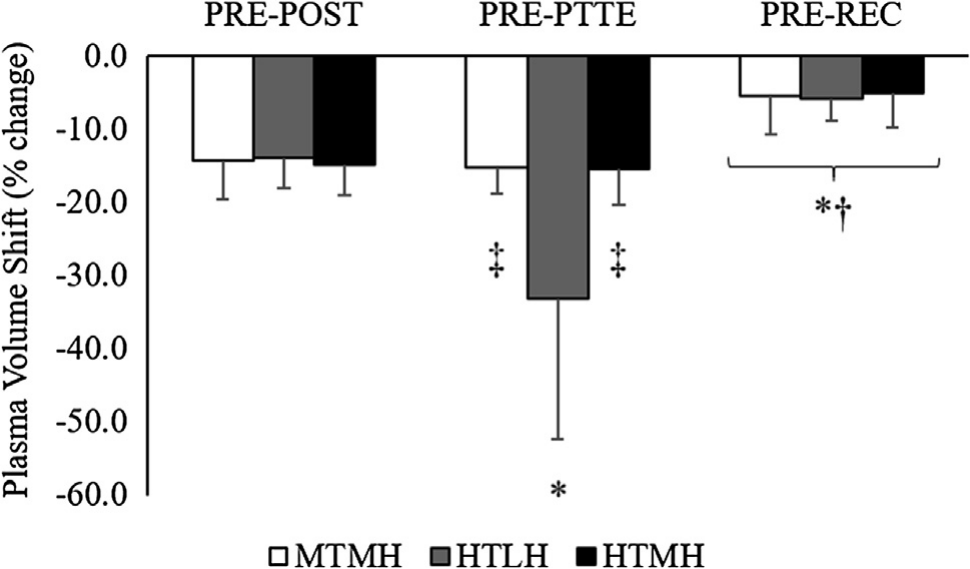
Plasma Volume Fluid Shifts. *Significant difference from PRE-POST during corresponding trial. † Significant difference from PRE-PTTE during corresponding trial. ‡ Significant difference from HTLH. Data are presented as mean (SD).

Plasma volume shifts are depicted in Fig. 4. A significant trial × time interaction (F = 8.552, *p* = 0.011, η^2^_p_ = 0.461) was observed for plasma volume fluid shifts. During MTMH, plasma volume decreased significantly more from PRE to POST and from PRE to PTTE compared to the shift from PRE to REC (*p’s* < 0.001). During HTLH, plasma volume decreased significantly more from PRE to PTTE compared to PRE to POST (*p* = 0.008) and PRE to REC (*p* < 0.001). Plasma volume also decreased significantly more from PRE to POST compared to PRE to REC (*p* < 0.001). During HTMH, plasma volume decreased significantly more from PRE to POST and from PRE to PTTE compared to the shift from PRE to REC (*p’s* < 0.001). Plasma volume decreased significantly more from PRE to PTTE during HTLH compared to MTMH (*p* = 0.008) and HTMH (*p* = 0.025).

### 3.4 TNF-α, STNFR1, and STNFR2 concentration

**Fig. 5:**
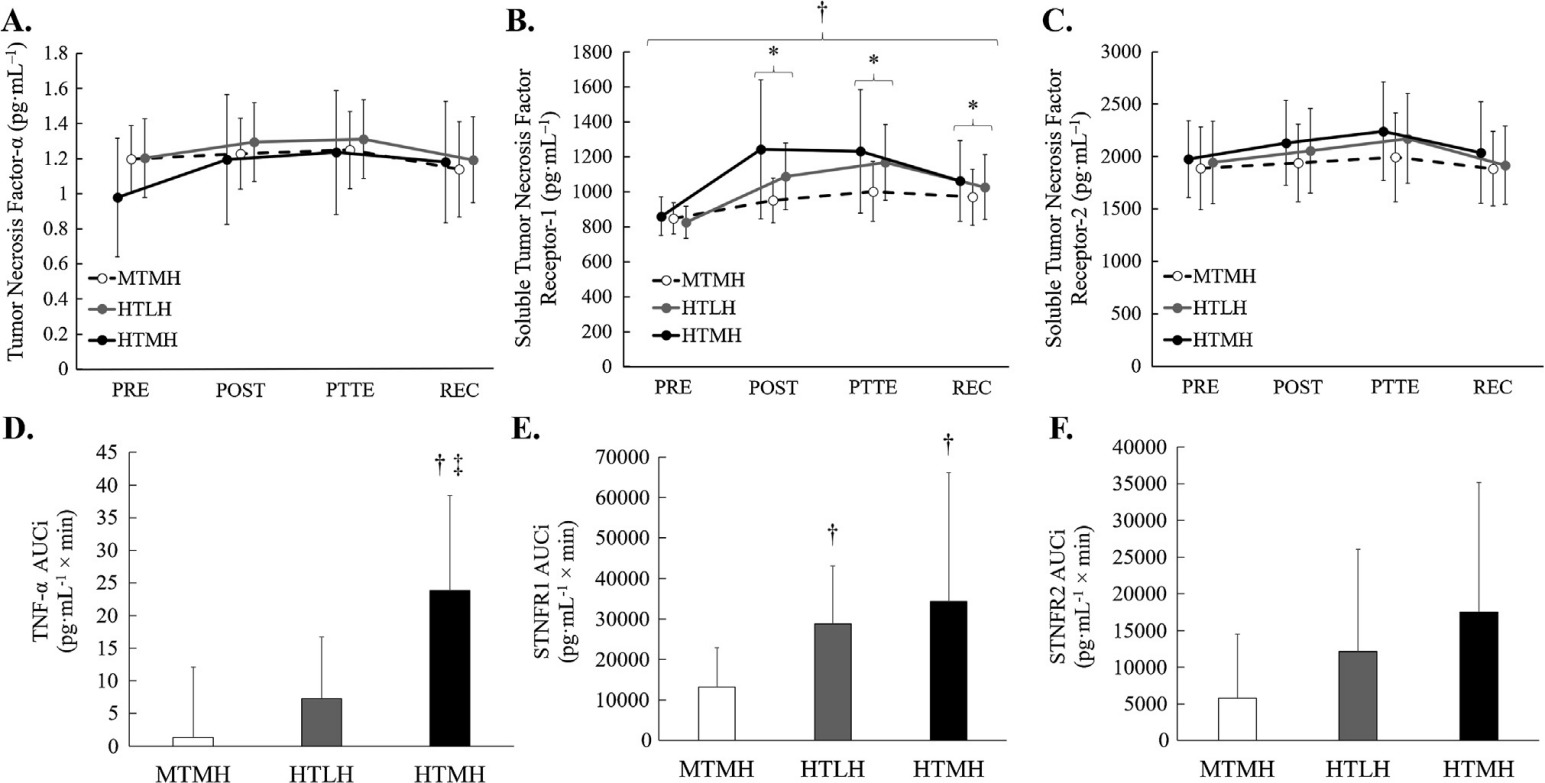
Changes in TNF-α and STNFRs. (A) Changes in plasma Tumor Necrosis Factor (TNF) -α concentrations. (B) Changes in soluble tumor necrosis factor receptor (STNFR) 1 concentrations. (C) Changes in STNFR2 concentrations. (D) AUCi analysis for TNF-α. (E) AUCi analysis for SNTNFR1. (F) AUCi analysis for STNFR2. Trials: HTLH, high temperature/low humidity; MTMH, moderate temperature/moderate humidity; HTMH, high temperature/moderate humidity. *Significant difference from PRE; main effect of time. † Significant difference from MTMH. ‡ Significant difference from HTLH. Data are presented as mean (SD).

Changes in plasma TNF-α concentrations are presented in Fig. 5. No significant trial × time interaction, main effect of time, or main effect of trial were observed for changes in TNF-α concentration. However, significant differences were noted between trials for TNF-α AUCi (F = 8.868, *p* = 0.001, η^2^_p_ = 0.446) with significantly greater AUCi observed during HTMH compared to MTMH (*p* = 0.008) and HTLH (*p* = 0.014).

Changes in plasma STNFR1 and STNFR2 concentrations are also presented in Fig. 5. No significant trial × time interaction was observed for changes in plasma STNFR1 concentrations; however, a significant main effect of time (F = 12.813, *p* < 0.001, η^2^_p_ = 0.243) and main effect of condition (F = 6.453, *p* = 0.002, η^2^_p_ = 0.097) were observed. When collapsed across time, STNFR1 concentration was significantly lower during MTMH compared to HTMH (*p* < 0.001) but not compared to HTLH (*p* = 0.055). With all trials combined, STNFR1 concentration increased from PRE to POST (*p* < 0.001), PTTE (*p* < 0.001), and REC (*p* = 0.001). STNFR1 also decreased from PTTE to REC (*p* = 0.025). A main effect of trial was noted for STNFR1 AUCi (F = 4.816, *p* = 0.039, η^2^_p_ = 0.304) with significantly lower AUCi observed during MTMH compared to HTMH (*p* = 0.026) and HTLH (*p* = 0.001). No significant trial × time interaction, main effect of time, or main effect of trial were observed for changes in STNFR2 concentration. Additionally, no significant difference was observed between trials for STNFR2 AUCi.

Since target receptors respond to absolute concentrations of the ligand in circulation rather than ligand production [38], circulating marker concentrations were not corrected for plasma volume shifts.

### 3.5 TNFR1 and TNFR2 surface expression on monocytes

**Fig. 6:**
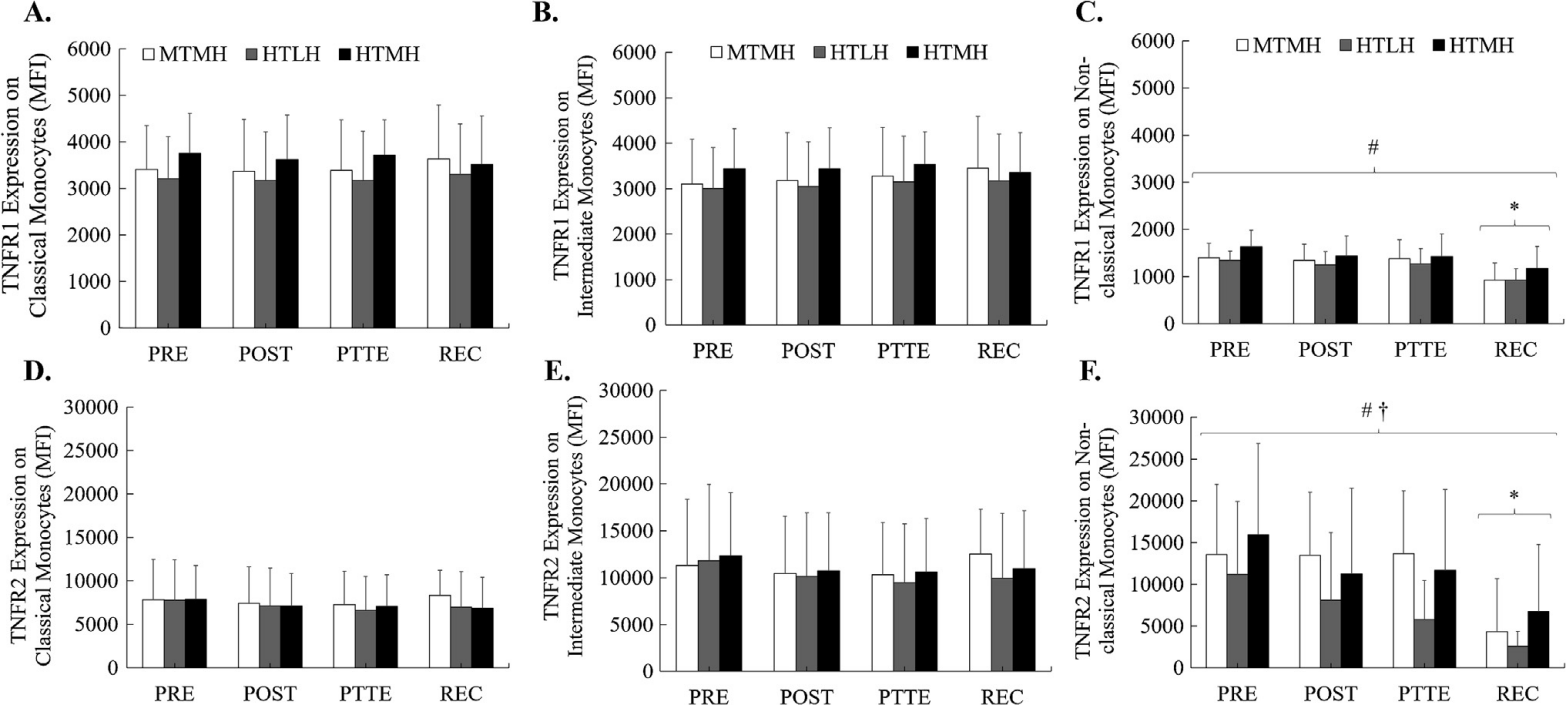
Changes in TNFR1 and TNFR2 Expression on Monocyte Subsets. (A) Changes in surface expression of tumor necrosis factor receptor (TNFR) 1 on classical monocytes. (B) TNFR1 on intermediate monocytes. (C) TNFR1 on non-classical monocytes. (D) TNFR2 on classical monocytes. (E) TNFR2 on intermediate monocytes. (F) TNFR2 on non-classical monocytes. *Significant difference from PRE; main effect of time. † Significant difference between MTMH and HTLH. # Significant difference between HTLH and HTMH. Data are presented as mean (SD).

Changes in surface expression of TNFR1 and TNFR2 on monocyte subsets are depicted in Fig. 6. No significant trial × time interaction, main effect of time, or main effect of trial were observed for changes in TNFR1 or TNFR2 expression on classical monocytes. Similarly, no significant changes in either receptor were observed on intermediate monocytes.

No significant trial × time interaction was observed for changes in TNFR1 expression on non-classical monocytes; however, a significant main effect of trial (F = 3.196, *p* = 0.046, η^2^_p_ = 0.072) and main effect of time (F = 7.109, *p* < 0.001, η^2^_p_ = 0.204) were observed. When collapsed across time, TNFR1 expression on non-classical monocytes was greater during HTMH compared to HTLH (*p* = 0.016). With all trials combined, TNFR1 expression on non-classical monocytes was lower at REC compared to PRE (*p* < 0.001), POST (*p* = 0.002), and PTTE (*p* = 0.001).

Similarly, no significant trial × time interaction was observed for changes in TNFR2 expression on non-classical monocytes; however, a significant main effect of trial (F = 3.176, *p* = 0.047, η^2^_p_ = 0.071) and main effect of time (F = 5.195, *p* = 0.002, η^2^_p_ = 0.158) was observed. When collapsed across time, TNFR2 expression on non-classical monocytes was lower during HTLH compared to both MTMH (*p* = 0.037) and HTMH (*p* = 0.028). With all trials combined, TNFR2 expression on non-classical monocytes was lower at REC compared to PRE (*p* < 0.001), POST (*p* = 0.008), and PTTE (*p* = 0.016).

## 4 Discussion

The purpose of this investigation was to compare the effects of aerobic exercise in three different environmental conditions (MTMH, HTMH, and HTLH) on changes in plasma concentrations of TNF-α, STNFR1, and STNFR2, as well as surface expression of TNFR1 and TNFR2 on monocyte subsets. The results of the present study suggest that exercise in the heat promotes elevations in TNF-α and STNFR1 compared to exercise in a moderate temperature. Moreover, TNFR1 expression on non-classical monocytes was elevated during the moderate humidity heat trial while TNFR2 expression on non-classical monocytes was lowest during the low humidity trial. Taken together, these findings suggest that exercise in the heat promotes increases in TNF-α and STNFR1 concentration. Furthermore, non-classical monocyte expression of TNFRs are impacted by temperature and humidity during exercise.

Studies investigating the effects of exercise in the heat on cytokine concentrations are limited. A study by Rhind and colleagues [12] evaluated the effects of a 40 min cycling trial at 65% VO_2_peak while the participants were immersed to mid-chest in either hot (39 °C) or cold (18 °C) water on cytokine responses and found significant increases in TNF-α concentrations following the hot condition only. Water immersion served as a “thermal clamp” wherein the hot condition exaggerated the exercise-induced rise in core temperature while the cold condition prevented it. Therefore, although the exercise intervention used by Rhind et al [12] was shorter in duration than the present study, the water immersion prevented the physiological mechanisms of heat loss that attenuate hyperthermia—such as evaporative heat loss via sweating [39]. As a result, the hot condition implemented by Rhind et al [12] elicited slightly larger increases in core temperature compared to the HTLH and HTMH conditions in the present study. Thus, increases in TNF-α concentration following exercise in the heat may be dependent on the rise in core temperature. Accordingly, TNF-α AUCi was higher during HTMH compared to both MTMH and HTLH, which is expected as HTMH also led to greatest rise in core temperature.

Previous studies have reported significant increases in circulating TNF-α concentrations following prolonged cycling in the heat [3], [13], while the present study did not observe time or trial effects on changes in TNF-α concentration following exercise. However, the aforementioned studies included endurance-trained men with mean aerobic capacities of 4.8 ± 0.3 L·min^−1^ [13] and 4.7 ± 0.4 L·min^−1^ [3] while the present study evaluated recreationally active men (3.9 ± 0.4 L·min^−1^) whom likely would not have tolerated a more rigorous exercise bout. As a result, the exercise intervention in the present study had a shorter duration and/or a lower intensity. To this end, TNF-α concentrations during the hot conditions in the present study were lower compared to those reported by Peake et al [13] and Starkie et al [3] during their heat trials. Given that both studies used the same ELISA kit to measure TNF-α concentrations as the present study, the discrepancy in TNF-α concentrations is likely a result of the differing exercise interventions. Therefore, 60 min of cycling at 60% VO_2_max in recreationally active men may not be a sufficient stimulus to elicit acute increases in plasma TNF-α concentrations despite the addition of heat stress. Notwithstanding, TNF-α AUCi, which represents the overall change in TNF-α concentration in response to the intervention, was significantly higher during HTMH compared to MTMH and HTLH. Given that core temperature at POST was higher during HTMH compared to both MTMH and HTLH, the TNF-α response to exercise is likely driven by changes in core temperature.

The results of the present study also suggest that exercise in the heat leads to increased plasma concentrations of STNFR1, but not STNFR2, when compared to exercise in moderate temperature. This is this first study, to our knowledge, to evaluate the effects of heat stress during exercise on changes in STNFR concentrations. STNFR1 and STNFR2 concentrations have been shown to be significantly elevated following prolonged endurance exercise (e.g. marathon race) [40]. Experimental endotoxemia in humans has been shown to result in downregulation of TNFR1 and TNFR2 expression on circulating monocytes [41]. This decline in TNFR expression was caused by endogenously produced TNF-α. Van der Poll and colleagues [41] proposed that this decline in TNFR expression may represent a mechanism to protect the host against excessive TNF-α activity, which can lead to tissue injury. Therefore, increased STNFR1 concentrations following exercise may indicate preferential ectodomain shedding of TNFR1 to prevent excessive TNF-α signaling.

Neither exercise nor environmental condition had an effect on surface expression of TNFR1 and TNFR2 on classical or intermediate monocytes. However, in non-classical monocytes, expression of both receptors was blunted 1 h following exercise (REC) relative to all other timepoints. Studies investigating the effects of exercise on changes in monocyte TNFR expression are limited to resistance exercise interventions. It was previously shown that TNFR1 expression on CD14^+^ monocytes is elevated 30 min following an acute bout of heavy resistance exercise [42], [43]. Similarly, Wells and colleagues [31] reported a significant increase in TNFR1 surface expression on intermediate (CD14^++^CD16^+^) monocytes one-hour following resistance exercise. Thus, monocyte TNFR expression in response to resistance exercise is distinct from the observations of this investigation following aerobic exercise. Notably, these resistance exercise protocols also resulted in elevated circulating concentrations of indirect muscle damage markers such as creatine kinase [43] and myoglobin [31], which suggests that increased TNFR1 expression on CD14^+^ monocytes may be in response to exercise-induced muscle damage.

Moreover, TNFR1 expression on non-classical monocytes was elevated during HTMH compared to HTLH and TNFR2 expression on non-classical monocytes was elevated during both MTMH and HTMH compared to HTLH. Thus, ambient relative humidity during exercise may have an effect on monocyte expression of TNFRs. Although TNFR expression on monocyte subpopulations have been previously shown to vary [17], whether monocyte subset expression of TNFRs responds differently to various stimuli is unknown. It was recently found that non-classical monocytes are senescent cells—viable and metabolically active cells that can no longer proliferate—and that the pro-inflammatory nature of this subset may be a manifestation of the senescence-associated secretory phenotype [44]. Thus, the decrease observed in TNFR1 and TNFR2 expression on non-classical monocytes may serve as a protective mechanism against TNF-α toxicity by limiting TNF-α signaling in this pro-inflammatory monocyte subset. This is further supported by the increase observed in STNFR1 concentration, which indicates a potential increase in ectodomain shedding of TNFR1 [33]. TNFR shedding can be induced by TNF-α as well as other inflammatory mediators, such as IL-6 and IL-1β [45]. Although a concomitant increase in STNFR2 was not observed, the decrease in TNFR2 expression may be due to ligand-induced internalization, which also serves as a negative feedback mechanism to limit TNF-α signaling [9]. This is the first study, to our knowledge, to measure TNFR expression on monocyte subsets following aerobic exercise in different environmental conditions. Further research is needed to elucidate the role of TNFRs on monocyte subsets in response to aerobic exercise and heat stress.

It is important to distinguish between the rise in core temperature that accompanies a fever, where endogenous pyrogens such as TNF-α, IL-1, and interferon increase the temperature set-point [46], and the rise in core temperature that results from heat exposure and physical exertion, where the set-point remains the same, but body heat is not effectively dissipated [47]. Previous studies reporting exercise-induced increases in TNF-α have failed to identify the mechanisms behind this finding [3], [13]. Notwithstanding, Rhind et al [12] proposed that even moderate exertional hyperthermia, as observed in the HTMH trial, may compromise the integrity of the gut mucosal barrier, allowing small amounts of LPS into the systemic circulation, and inducing TNF-α production [48]. Therefore, it is plausible that the observed TNF-α response may have been triggered by mild endotoxemia.

One of the limitations of this study was that the aerobic exercise protocol employed was not sufficiently robust to elicit a substantial increase in plasma TNF-α when compared to previous studies [3], [13]. A more prolonged intervention likely would have led to a more pronounced TNF-α response and more pronounced changes in STNFRs and monocyte expression of TNFRs. Furthermore, TNFR expression was only observed in circulating monocyte subpopulations. Since TNFR1 is constitutively expressed in nearly all cell types [6], the origin of the rise in STNFR1 concentration is unknown and its relationship to the decrease observed in TNFR1 expression on non-classical monocytes is speculative. Lastly, circulating marker concentrations were not adjusted for changes in plasma volume following exercise due to the importance of molar exposure to the receptors. Therefore, the increases observed in TNF-α and STNFR1 may be a result of decreased plasma volume rather than increased biosynthesis and ectodomain shedding, respectively. However, plasma volume changes were similar between HTMH and MTMH and therefore the differences observed in TNF-α and STNFR1 cannot be attributed solely to plasma volume changes.

## 5 Conclusions

The results of this study indicate an acute bout of moderate-intensity cycling in a high ambient temperature in recreationally active Caucasian men results in a modest increase in circulating concentrations of TNF-α and STNFR1 when compared to exercise in a moderate temperature. Athletes and individuals in occupations that require physical exertion during heat exposure should aim to limit the rise in core temperature to protect from inflammation-induced injury and immunosuppression [24]. Furthermore, TNFR1 and TNFR2 expression on non-classical monocytes is down-regulated one hour post-exercise and increased ambient relative humidity may blunt surface expression of TNFR2 on non-classical monocytes. These findings add to our knowledge of the inflammatory events that follow aerobic exercise in hot climates.

## Grants

This study was partially funded by the Kent State University Research Council and the Kent State University College of Education, Health and Human Services SEED Award.

## CRediT authorship contribution statement

**Eliott Arroyo:** Writing - original draft, Investigation, Formal analysis, Visualization. **Joseph A. Laudato:** Investigation, Writing - review & editing. **Brandon M. Gibson:** Investigation, Writing - review & editing. **Cody S. Dulaney:** Investigation. **Jeremiah A. Vaughan:** Investigation, Project administration. **Brittany N. Followay:** Investigation, Project administration. **Ellen L. Glickman:** Conceptualization, Methodology, Resources, Supervision, Project administration, Funding acquisition. **Adam R. Jajtner:** Writing - review & editing, Investigation, Formal analysis, Conceptualization, Methodology, Resources, Supervision, Project administration, Funding acquisition.

## Declaration of Competing Interest

The authors declare that they have no known competing financial interests or personal relationships that could have appeared to influence the work reported in this paper.
